# Methicillin-resistant *Staphylococcus aureus* colonization among medicine and health science students, Arba Minch University, Ethiopia

**DOI:** 10.1038/s41598-022-14212-y

**Published:** 2022-06-17

**Authors:** Ermiyas Mekuriya, Aseer Manilal, Addis Aklilu, Melat Woldemariam, Tadiwos Hailu, Biresaw Wasihun

**Affiliations:** 1 Department of Medical Laboratory Science, College of Medicine and Health Sciences, Arba Minch University, Arba Minch, Ethiopia; 2 School of Medicine, College of Medicine and Health Sciences, Arba Minch University, Arba Minch, Ethiopia; 3 Department of Midwifery, College of Medicine and Health Sciences, Injibara University, Injibara, Ethiopia

**Keywords:** Microbiology, Antimicrobials, Bacteriology

## Abstract

Individuals with Methicillin-Resistant *Staphylococcus aureus* (MRSA) colonized nasal cavities were at greater risk of developing the infection and can serve as potential reservoirs of transmission. Aim of this study is to determine the extent of nasal carriage and associated factors linked to MRSA in medical and health science students of Arba Minch University (AMU), Ethiopia, who are much prone. An institution based cross-sectional study was conducted at AMU from 01st August through 30th November, 2020 by means of a systematic sampling technique using a structured questionnaire. Nasal swabs samples were collected and *S. aureus* were identified following standard microbiological methods. Methicillin resistance was tested using cefoxitin disk and antimicrobial susceptibility tests were performed by Kirby-Bauer disk diffusion. Biofilm forming ability was phenotypically detected by micro-titer plate assay. Descriptive statistics and multivariable logistic regression analysis were done by Statistical Package for Social Service (SPSS) version 25. The overall prevalence of *Staphylococcus aureus* and MRSA were 27.1% (70/258) and 7.4% (19/258) respectively. Methicillin-Resistant *S. aureus* carriage were higher among medical interns, 16.9% (11/65). Isolates in general were co-resistant to antibiotics, such as trimethoprim-sulfamethoxazole (63.2%) and tetracycline (48.4%). Multidrug resistance (MDR) were observed among 52.6% (10/19) of the isolates. Besides, 31.4% (6/19) of MRSA were biofilm producers and all of them were MDR. Multivariable analysis showed that mean exposure for > 2 years to hospital settings [*p* = 0.048, AOR: 4.99, 95% CI 1.01–24.66] and the habit of sharing clothing and sports equipment [*p* = 0.017, AOR: 5.43, 95% CI 1.35–21.83] were statistically significant. The overall prevalence of nasal colonized MRSA among students were comparatively lower than that observed in other studies done in Ethiopia itself. An alarming factor is that, 60% of MDR-MRSA were biofilm producers.

## Introduction

Methicillin-resistant *Staphylococcus aureus* includes any strain of *S. aureus* that has developed resistance to β-lactam antibiotics. The organism acquires resistance via the incorporation of a mecA gene into its chromosome at a specific site; mecA encodes an alternative penicillin-binding protein that has low affinity towards semisynthetic penicillins, including methicillin, nafcillin, and oxacillin agents^1^.

Now a days MRSA is a major threat to the public health, in countries where HIV/AIDS, malaria, tuberculosis, malnutrition, crowded living, change in climate contribute to an increased risk of *Staphylococcal* infections^2^. Methicillin-resistant *S. aureus* has been categorized into three main types, namely healthcare-acquired, community and livestock-associated based on its involvement, and can result in nosocomial, community and livestock-associated infections respectively^3^.

Colonization by MRSA is a well-known risk factor in developing infections both in adults and children. This is especially true in the case of patients who acquire MRSA from hospital settings where the risk of developing an infection is around 30%^4^. Strains of MRSA are usually spread by direct skin-to-skin contacts and may occur during hospital admissions, transfer, or other healthcare-related contacts; however, the roles of shared public spaces (e.g., dormitories, gym, barracks, etc.) were also noted^5^. It is reported that close contacts with a person already colonized or infected with MRSA can result in a 7.5-fold greater risk of becoming colonized. Persons colonized with MRSA are an increased risk of developing the infection and can serve as a source of transmission^6^.

The nose (anterior nasal mucosa) is known to be the primary ecological reservoir of MRSA in humans and is a main risk factor causing infections in various clinical settings. Several surfaces in hospital environments, gowns of healthcare workers, and patient-care items contaminated or colonized with MRSA have been shown to contribute to the risk of transmission^7^. Since medical students have long period of hospital stay, it is envisaged that they are also at greater risk of nasal colonization by MRSA, as in the case of health care workers.

Biofilm formation is an important virulence factors. Large biofilm-like bacterial aggregates including *S. aureus* and other species were found in the mucosal samples taken from patients with chronic rhino-sinusitis^8^. In addition, a recent study done in Arba Minch reported that biofilm forming nasal isolates of MRSA were more resistant than their non-biofilm-forming counterparts^9^.

As healthcare workers are commonly exposed to patients in their work set-up, it is important to assess the rate of prevalence of MRSA carriage to evaluate the extent of preventive and control measures to be taken, which can limit the spread. Accurate susceptibility testing is essential to be formulated much in advance of selecting empirical treatment strategies in the case of an unexpected outbreak. Different surveillance policies for detection of carriage and/or decolonization have been proven to be effective in minimizing the rate of infections. Elucidation of the prevalence of MRSA carriage and studies on the profiles of antibiotic susceptibility are pivotal. However, all over Africa, there exists no strict surveillance system or an effective control program to deal MRSA, which further worsen the menace.

As of now, only limited number of studies are carried out in the national level regarding the nasal carriage of MRSA, especially among medical and health science students. Furthermore, research on the ability of MRSA to form biofilm is not done extensively. Therefore, the present study is aimed at determining the extent of nasal carriage of MRSA, its antibiotics susceptibility, ability to form biofilm and associated factors, among the medical and health science students of AMU.

## Results

### Socio-demographic characteristics

A total of 258 participants were enrolled with a response rate of 100%. Of these, 72.9% (*n* = 188) were males. The age of study participants ranged from 21 to 27, with a mean value of 23 ± 1.13. With regard to the proportion of health science students among the total participants, 10.8% (*n* = 28) were from the department of nursing, 10.5% (*n* = 27) from health officers, 8.9% (*n* = 23) from midwifery, 8.5% (*n* = 22) from medical laboratory, 4.7% (*n* = 12) from anesthetic, and 4.3% (*n* = 11) from radiology. The study population is re-grouped into three categories such as medical interns, clinical and health science students for the convenience of statistical analysis (Table1).

**Table 1:** Socio-demographic, clinical and behavioral characteristics of medicine and health science students of Arba Minch University, Arba Minch, southern Ethiopia, 2020.

Variables		Frequency	Percentage (%)
Sex	Male	188	72.9
	Female	70	27.1
Age	Less than 24	172	66.6
	24 and above	86	33.3
Category of students	Clinical and OHS students	193	74.8
	Medical intern	65	25.2
Mean exposure to hospital	Less than 6 months	98	38.0
	6 months to 1 year	60	23.3
	1 to 2 years	35	13.6
	Greater than 2 years	65	25.1
Nose picking habit	Yes	125	48.4
	No	133	51.6
Use of gloves while handling patients	Always	63	24.4
	Sometimes	140	54.3
	Rarely	55	21.3
Antibiotic use (last three months)	Yes	13	5.0
	No	245	95.0
Ways of antibiotic usage	Full course	257	99.6
	Incomplete course	1	0.4
Visiting gymnasium	Yes	64	24.8
	No	194	75.2
Sharing of clothing	Yes	65	25.2
	No	193	74.8
Participate in invasive procedures	Yes	150	58.1
	No	108	41.9

### Clinical and behavioral characteristics

Out of 258 participants, 25.1% (*n* = 65) were medical interns and had exposure for more than 2 years in the hospital, 54.3% (*n* = 140) of them use gloves sometimes while handling patients; 51.6% (*n* = 133) are devoid of the habit of nose picking. On the other hand, 24.8% (*n* = 64) participate in sports or visit gymnasium. About 58% (*n* = 150) had indulged in invasive procedures and 58.9% (*n* = 152) of them were accommodated in a single dormitory type meant for a group of 6 members, and 69.8% (*n* = 180) clean their dormitory twice a week. Most of the participants, ie., 78.7% (*n* = 203) maintain only moderate level of hand sanitization practices (Table1).

### Prevalence of nasal *S. aureus* carriage

During the study, totally 70 (27.1%) isolates of *S. aureus* were recovered from 258 participants. The rate of nasal carriage was relatively high among females in terms of percentage (32.9%). Nasal carriage of *S. aureus* was detected among 29 out of 86 (33.7%) members from the age group of 24 and above and also it was the highest among medical interns, ie., 26 (40%) followed by clinical students, 17 (24.3%) and other health science students, 27 (22%) (Table 2).

**Table 2:** Prevalence of *S. aureus* and MRSA among medicine and health science students of Arba Minch University, Arba Minch, southern Ethiopia, 2020.

Variables		*S. aureus* carriage		MRSA carriage		Total
		Yes (*n* = 70)	No (*n* = 188)	Yes (*n* = 19)	No (*n* = 51)	
		*n* (%)				
Sex	Male	47 (25)	141 (75)	10 (5.3)	178 (94.7)	188
	Female	23 (32.9)	47 (67.1)	9 (12.9)	61 (87.1)	70
Age	Less than 24	41 (23.8)	131 (76.2)	8 (4.7)	164 (95.3)	172
	24 and above	29 (33.7)	57 (66.3)	11 (12.8)	75 (87.2)	86
Category of students	Medical intern	26 (40)	39 (60)	11 (16.9)	54 (83.1)	65
	Clinical student	17 (24.3)	53 (75.7)	3 (4.3)	67 (95.7)	70
	Other health science	27 (22)	96 (78)	5 (4.0)	118 (96.0)	123

### Prevalence of nasal MRSA carriage

**Figure 1:**
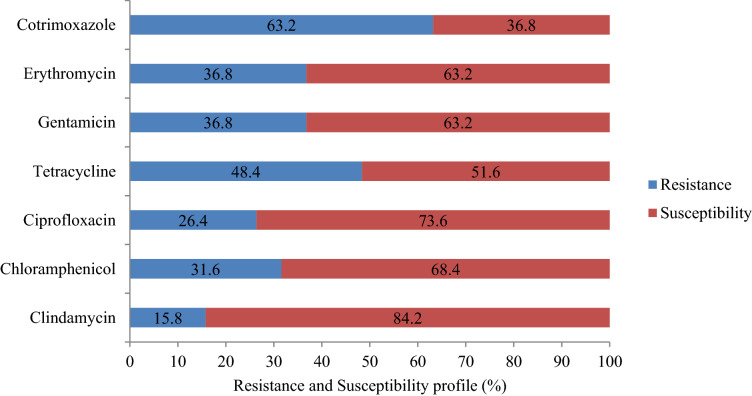
Antimicrobial susceptibility profiles of nasal MRSA isolates among Medicine and Health Science Students of Arba Minch University, Arba Minch, southern Ethiopia, 2020.

Among the 70 isolates of *S. aureus,* 19 were identified as MRSA, and hence, the prevalence among *S. aureus* positive participants was 27.1%. Overall prevalence of MRSA was 7.4% (*n* = 19); the carriage was found to be higher among medical interns, ie., 16.9% (*n* = 11) followed by clinical year students, 4.3% (*n* = 3) and health science students, 4.0% (*n* = 5). Of the total MRSA carriage, males account for the 53% (*n* = 10); carriage was found higher, ie., 12.8% (*n* = 11) among the age group of 24 and above (Table 2; Fig. 1).

### Antibiotic susceptibility tests

**Figure 2:**
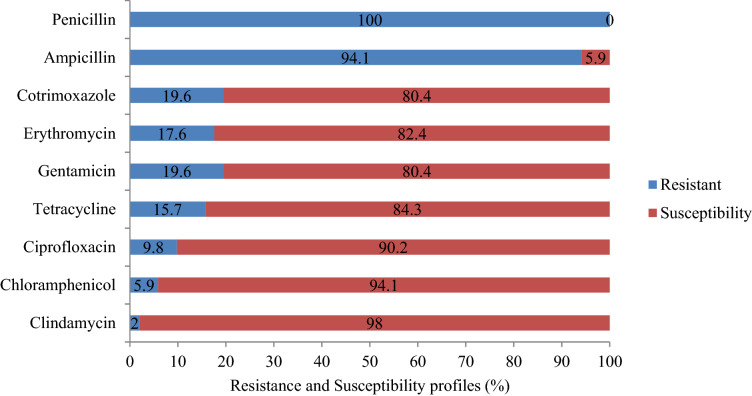
Antimicrobial susceptibility profiles of nasal MSSA isolates among Medicine and Health Science Students of Arba Minch University, Arba Minch, southern Ethiopia, 2020.

A total of 51 methicillin susceptible *S. aureus* (MSSA) isolates were subjected to tests against nine antibiotics. It was found that majority of these isolates were susceptible to seven antibiotics ie., to an extent of 80.4 to 98.0%. At the same time, they showed a very high level of resistance to two antibiotics such as penicillin (100%) and ampicillin (94.1%) (Fig. 2). Most of the isolates of MRSA were found susceptible to antibiotics such as clindamycin (84.2%), ciprofloxacin (73.6%) and chloramphenicol (68.4%). It was found that 63.2% of each of the isolates were susceptible to gentamicin and erythromycin whereas 52.6% showed susceptibility to tetracycline. On the other hand, 63.2% of the isolates were resistant to trimethoprim-sulfamethoxazole (Fig. 1). No intermediate category was observed in this study. Of the nine erythromycin resistant isolates of MSSA, erythromycin inducible clindamycin resistance (iMLSB) was detected in 55.6% (*n* = 5). Among the isolates of MRSA, iMLSB phenotype was observed in 57.1% (*n* = 4) of erythromycin resistant cases**.**

### Multi-drug resistance profiles of MSSA and MRSA

Among the isolates of MSSA, 39.2% (*n* = 20) were MDR; of them, 65% (*n* = 13) showed resistance to three different classes of antibiotics. About 35% (*n* = 7) isolates showed resistance to four classes of antibiotics in varying profiles. It was found that ten of the MRSA isolates are MDR (52.6%), and out of these, seven isolates (70%) showed resistance to three classes of antibiotics. Two isolates displayed resistance to four classes of antibiotics, ie., CLN/CHL/TET/GEN. Besides, one isolate of MRSA showed resistance to five classes of antibiotics (Table 3).

**Table 3:** Multidrug resistance profile of nasal MRSA isolates among Medicine and Health Science Students of Arba Minch University, Arba Minch, southern Ethiopia, 2020.

Resistance profile	MDR	Resistance profiles	Source of isolate			No. of isolates *n* (70)
			MI	CS	OH	
MRSA						
	R3	E/ SXT /TET	2	1	1	4
	R3	E/CLN/TET	2	1	–	3
	R4	CLN/ CHL/TET/GEN	1	–	1	2
	R5	E/TET/CLN/GEN/CPR	1	–	–	1
Total MRSA			6	2	2	10/19(52.6)
MSSA	R3	AMP/PEN/GEN	2	1	1	4
	R3	AMP/PEN/SXT	3	1	1	5
	R3	AMP/PEN/TET	2	1	1	4
	R4	AMP/PEN/E/SXT	2	1	1	4
	R4	AMP/PEN/GEN/CLN	2	–	1	3
Total			11	4	5	20/51(39.2)

PEN: penicillin, CPR: ciprofloxacin, CLN: clindamycin, GEN: gentamicin, E: erythromycin, CHL: chloramphenicol, AMP: ampicillin, TET: tetracycline and SXT: trimethoprim-sulfamethoxazole, MI: Medical intern, CS: clinical students, OH: other health students, R3: resistant to 3 antibiotics, R4: resistant to 4 antibiotics, R5: resistant to 5 antibiotics.

### Biofilm forming potentials of MRSA

According to the OD reading from micro-titer plates, 2 (10.5%) isolates exhibited strong biofilm forming potency followed by another couple of isolates that displayed only a moderate level of activity. A third pair of isolates was found to be weak biofilm formers. On the other hand, 13 (68.5%) isolates were biofilm non-formers. In total, biofilm forming isolates were 31.6%, (6/19). All the six isolates of biofilm formers were obtained from medical interns and 60% of them were found to be MDR.

### Factors associated with MRSA carriage

Participants in the age group of 24 and above were colonized with MRSA in a relatively higher proportion than others, however was not statistically significant. The carriage was found to be higher among females, 39.1% (*n* = 9) and is statistically significant (*p* = 0.04). Among the 19 participants tested positive for MRSA, 13 practiced sports or visited gymnasium, but this also was not statistically significant (*p* = 0.48). In bivariable analysis, MRSA colonization was found to be statistically significant with variables such as sex (*p* = 0.046), age (*p* = 0.023), category of students (*p* = 0.002), mean exposure to hospital (*p* = 0.002), habit of nose picking (*p* = 0.010), usage of gloves during the handling of patients (*p* = 0.000), sharing of clothing and sports equipment (*p* = 0.000), participation in invasive procedures (*p* = 0.162), strength of accommodation in a single dormitory (*p* = 0.010) and the hand sanitization level (*p* = 0.000) (Table 4). However, the results of multivariable analysis showed that odds of having a nasal MRSA are 4.9 times higher in students having > 2 years of mean exposure to hospital [*p* = 0.048, AOR: 4.99, 95% CI 1.01–24.66]. Besides, the odds of MRSA colonization are 5.13 times higher in participants having a habit of nose picking [*p* = 0.05, AOR: 5.13, 95% CI 0.94–27.74)]. Similarly, the odds of being colonized by MRSA was 5.4 times higher in students who share clothing and sports equipment [*p* = 0.017, AOR: 5.43, 95% CI 1.35–21.83].

**Table 4:** Bivariable and multivariable logistic regression analysis of factors associated with nasal carriage of MRSA among Medicine and Health Science Students of Arba Minch University, Arba Minch, southern Ethiopia, 2020.

Variables	Category	MRSA carriage		Analysis			
		No	Yes	COR (95% CI)	*p* value	AOR (95% CI)	*p* value
Sex	Male	188	10	1	1	1	
	Female	70	9	2.62 (1.01–6.76)	0.04*	3.24(0.60–17.53)	0.17
Age	Less than 24	172	8	1	1	1	
	24 and above	86	11	3.00(1.16–7.78)	0.02*	0.82(0.16–4.01)	0.81
Category of students	Medical Interns	65	11	4.71(1.80–12.30)	0.002*	4.78(0.65–34.69)	0.12
	Clinical and OHS students	193	8	1	1	1	
Mean exposure to hospital	≤ 2 years	193	8	1	1	1	
	> 2 years	65	11	4.71(1.80–12.30)	0.002*	4.99(1.01–24.66)	0.04**
Habit of nose picking	Yes	125	15	4.39(1.41–13.64)	0.01*	5.13(0.94–27.74)	0.05**
	No	133	4	1	1	1	
Participation in sports	Yes	64	6	1.44(0.52–3.96)	0.48	–	–
	No	194	13	1	1		
Sharing of clothing and sports equipment	Yes	65	13	7.79(2.82–21.49)	0.000*	5.43(1.35–21.83)	0.01**
	No	193	6	1	1	1	
Participation in invasive procedures	Yes	150	14	2.12(0.74–6.07)	0.16*	1.61(0.21–11.97)	0.64
	No	108	5	1	1	1	
Use of glove while handling patients	Always	197	8	1	1	1	
	Sometimes	42	11	6.44 (2.44–17.01)	0.00	2.45 (0.76–7.82)	0.13

*Statistically significant at *p* < 0.25 in bivariable analysis; **Statistically significant at *p* < 0.05 in multivariable analysis, AOR: Adjusted odds ratio, COR: Crude odds ratio, 1: reference group, CI: Confidence interval.

## Discussions

During the last three decades, MRSA has evolved as one of the most important causes of hospital acquired infection worldwide. It is a well acknowledged fact that nasal colonized healthcare workers in hospitals can become reservoirs leading to the spread of MRSA to susceptible patients^15^. Its identification can help in the initiation of decolonization measures combined with other precautions such as taking care of hand hygiene aimed at the reduction of transmission and spread.

In Ethiopia, nasal colonization by MRSA among medical and health science students are not studied extensively, despite their increased exposure to nosocomial pathogens from risky patients^16^. This innovative study provides a baseline data pertaining to the prevalence, associated factors, antimicrobial susceptibility profiles, and biofilm-forming potentials of nasally colonized MRSA among such a group in the Arba Minch town of Ethiopia. According to the present study, the overall prevalence of nasal *S. aureus* among medical students is 27.1%. This value is more or less in parity with the results of studies done among students from other countries such as Poland (30%)^17^, China (23.1%)^18^, Thailand (29.7%)^19^ and Saudi Arabia (25.3%)^20^. However, it is lower than that reported from a study done in Nepal (35%)^21^, which could be due to variations in geographical locations, study settings, methodology used and also because of the inclusion of undergraduates other than medical/health science students who had less frequent exposure to hospitals. Our results are showing a relatively upward trend in comparison to those studies reported from Jimma, Ethiopia (21.1%)^22^, Tanzania (21.0%)^23^, Nigeria (14%)^24^, and Iran (19.6%)^25^. These inconsistencies might be attributed to differences in situations existing in various countries and hospital settings and also in infection control and prevention policies and standard precautions taken. Moreover, the overall prevalence of MRSA found in this study is 7.4% and is comparable to the studies conducted in Jimma, Ethiopia (8.4%)^22^, and China (9.4%)^18^. However, this percentage is lower than the extent reported from Nepal (19.5%)^21^, Iran (13.14%)^25^, and Nigeria (13.6%)^26^. The lower prevalence found in our study might be due to the inequalities in the nature of environmental exposures, spatial differences and types of methodologies used.

Also the prevalence found in the current study is relatively higher than that previously reported from Tanzania (1.5%)^27^, Democratic Republic of Congo (2.6%)^28^, and Saudi Arabia (6.7%)^20^. These fluctuations might be due to the variations in infection control and prevention policies existing across countries, differences in perceptions, awareness of students about the epidemiology of MRSA and the nature of geographical status, as already cited above.

Another interesting finding is that the wider extent of *S. aureus* colonization (27.3%) and lower MRSA carriage (7.4%) observed in Arba Minch are comparable to an earlier study reported from Jimma, Ethiopia^22^ and could be due to the similarity in methodologies used. However, as mentioned earlier, the prevalence of MRSA among students was 7.3%, indicating a rate of nasal colonization more or less equivalent to that existing among the general population and healthcare workers at the national level^16^. In that meta-analytical study, authors arrived at a pooled prevalence of 10.94%, in the case of nasal MRSA. In the present study, the prevalence of nasal MRSA is found to be higher in medical interns compared to other students, however, there was no notable statistically significant association. Our results are in line with a couple of the earlier studies conducted in Jimma, Ethiopia^22^ and Saudi Arabia^20^. The high prevalence of nasal MRSA in medical interns could be attributed to their long-term exposure to hospital environments and regular interactions with patients. In this context, it is envisaged that medical students carrying multidrug-resistant *S. aureus* can pose serious threats to patients as well as to other people in their immediate vicinity. It is not possible to draw any strong conclusions from this findings and therefore further in-depth study is required to examine the relationship between MRSA nasal colonization in medical students and the subsequent transmission to patients.

The rise of drug-resistant MRSA is a serious challenge in the treatment and control of staphylococcal infections. Therefore, the rapid detection of MRSA and elucidation of its susceptibility profile are crucial from a treatment point of view, especially in the context of limited therapeutic regimens existing. Literature related to the antimicrobial susceptibility profiles of MRSA in medical students in Ethiopia is scanty. In this study, totally seven commonly used antibiotics were selected to inspect the susceptibility profiles of MRSA. Antimicrobial susceptibility profiles were detected in the case of all the MRSA isolates, but to varied extents. Resistance were particularly exhibited against the antibiotics, viz., trimethoprim-sulfamethoxazole (63.2%) (95% CI 47.8%, 78.5%) followed by tetracycline (48.4) (95% CI 27.1%, 64.6%), however, lower extents of resistance only were displayed against gentamicin and erythromycin, each 36.2% (95% CI 19.1%–49.3%) and chloramphenicol, 31.6% (95% CI 17.1%–45.2%).

Resistance exhibited against trimethoprim-sulfamethoxazole (63.2%) was in line with a couple of studies conducted in different regions of Ethiopia, such as Adigrat (64.3%)^29^ and Dessie (66.7%)^30^. However, it is lower compared to the data obtained from studies conducted in Poland (100%)^17^ and Jimma, Ethiopia (83.9%)^22^. These differences in resistance might be due to the types of antimicrobials used and the variations in the characteristics of study populations included.

In our study, 48.4% of isolates showed resistance to tetracycline and this is in contrast to the results of other works reported from Kenya (35%)^31^ and Poland (37.5%)^17^; but less severe than the extent of resistance found in a study done in Jimma, Ethiopia (64.5%)^22^. The resistance profile of MRSA against gentamicin (36.8%), erythromycin (36.8%) and chloramphenicol (31.6%) observed currently are in line with the results of a study conducted in Kenya^31^; but is less pronounced than that found in Adigrat, Ethiopia^29^.

On the other hand, isolates of MRSA showed only nominal resistance to clindamycin (15.8%). A similar trend of resistance was observed in a study reported earlier from Jimma, Ethiopia (16.1%)^22^. In the case of ciprofloxacin, enhanced resistance was reported from Jimma (51.6%)^22^, Adigrat^29^ and Mekelle^32^, compared to our results (26.4%). These discrepancies might be due to the fact that there is a gradual increase in antibiotic resistance which in turn might be the result of overuse or misuse of antimicrobial agents and or due to probable mutations and horizontal gene transfer occurring among bacteria.

Results revealed that out of the 19 MRSA isolates tested for inducible clindamycin resistance, 57.1% were positive and this is much elevated than a couple of studies done in Tanzania (31.8%)^6^ and Egypt 5.3%^33^. These variations might be due to the deviation in the trends in usage of antibiotics, methodology adopted, and also the type of study participants involved.

Multidrug resistance shown by MRSA is currently considered as a global threat by WHO. In the present study, MDR was observed in the case of 52.6% MRSA. Literature linked to MDR among medical students is limited, but this result is matching with a couple of studies conducted in Mekelle (50%)^32^ and Jimma (48.4%)^22^, Ethiopia; however, not that severe compared to the extent of MDR revealed by earlier studies conducted in Arba Minch itself, (75%)^34^, Egypt (85.8%)^33^ and Tanzania (72.7%)^6^. Surprisingly, results of another study conducted in Arba Minch previously showed a very weak level of MDR ie., 18.7% only^9^. The emergence of high MDR-MRSA could be due to the unscrupulous usage of those antibiotics in the study area. The overall picture of antimicrobial susceptibility profile obtained could be helpful for judiciously choosing antibiotics in future for the effective management of MRSA.

In the present study, 31.6% of the isolates produced biofilm and among them almost 33.4% were very effective producers of biofilm whereas some of them were moderate and weak i.e., 33.3% each. Published literature related to biofilm forming ability of MRSA found in medical students is limited in an Ethiopian context. Earlier studies performed in Arba Minch among HIV patients reported that 34.3% isolates of MRSA were biofilm formers^9^. However, this is lower than that found among food handlers (80%) in the same town^34^.

It has been found that 60% of MDR isolates among MRSA were biofilm formers and all these are isolated from medical interns. Further statistical analysis of the process of biofilm-formation by MRSA and MDR isolates revealed that it is positively skewed towards MDR isolates. This trend is comparable to that found in some of the previous studies conducted in Nigeria^35^, and Brazil^36^.

Different socio-demographic and behavioral associated factors had been analyzed by taking into consideration the spread of nasal carriages of MRSA among medicine and health science students. Of those factors, mean exposure to hospital for > 2 years was one of the significantly associated factors (*p* = 0.04) related to the nasal carriage of MRSA. It is an important predisposing factor connected to colonization in the population analyzed in this study. Here, the odds of having MRSA isolate was 4.9 times higher among students who have mean exposure to hospital for > 2 years. Other vital factor which are intimately associated with the nasal carriage of MRSA was sharing of clothing and sports equipment (*p* = 0.01). In this study, the odds of being colonized by MRSA was 5.4 times higher among students who share clothing and sports equipment than their counter parts.

## Conclusions

The prevalence of nasal carriage of *S. aureus* and MRSA in this study were found to be comparable to that reported earlier in Ethiopia itself. Higher MRSA carriage rate was noticed among medical interns in comparison to other health science and clinical students. Of the MRSA isolates, about 52.6% were considered as MDR and 60% of them were found to be biofilm formers. In addition, 84.2% of the MRSA isolates were susceptible to clindamycin. Students having > 2 years of mean exposure to hospital and those who share clothing and sports equipment were found to be significantly associated with the acquisition of nasal carriage of MRSA.

## Materials and methods

### Study area, design and period

An institution based cross sectional study was conducted in the College of Medicine and Health Sciences, AMU, southern Ethiopia from 01 August through 30th November 2020, located in Arba Minch town which enrolls 1598 students in total in the health sciences and medical programs (regular). In 2019/2020 academic year, among the new entrants, 1029(64.4%) were males. The campus consists of a number of departments such as medicine, nursing, pharmacy, anesthesia, midwifery, radiology, medical laboratory science, environmental health, health informatics and public health.

### Study population and eligibility criteria

All undergraduate students belonging to clinical medicine and health sciences, who had a clinical exposure for eight hours per day for more than 3 months in any hospital setting during the study period, are considered. The inclusion criteria set for the study comprises; 1. all medical and health science graduating class students who have exposure for 8 h per day for the previous 3 months and who are available during the study period; 2. students without a history of hospitalization in the preceding six months (i.e., they are not exposed because of admission, rather exposed during care giving). The exclusion criteria include; (1) participants who had nasal infections during the study period, (2) those who received intranasal antibiotic ointment or other antibiotics within the previous two weeks of the commencement of the study or who underwent a nasal decolonization procedure. The study protocol was approved by the Institutional Research Ethics Review Board of the College of Medicine and Health Science of Arba Minch University (IRB/464/12). This study was conducted in accordance with the declaration of Helsinki. Besides, separate permission was procured from Arba Minch University, College of Medicine and Health Sciences. Formal written consent was obtained from each study participant. Confidentiality was strictly maintained from sample collection up to the final report writing.

### Sample size determination and sampling technique

The sample size was calculated by using a single population proportion formula, where, *p* value of 50% was chosen due to the dearth of prior studies conducted among similar study population. Calculations were done using an open Epi version 3 by assuming 95% confidence level with 5% degree of precision, as followed in the case of sample size for frequency^10^.

**Table:** 

Population size (for finite population correction factor or fpc)(*N*):	535
Hypothesized % frequency of outcome factor in the population (*p*):	50% ± 5
Confidence limits as % , (absolute + /− %)(*d*):	5%
Design effect (for cluster surveys-*deff*):	1

Accordingly, the sample size determined was 224.

Then by assuming a non-response rate of 15%, the total subjects included in the study was 258 (*n* = 224; *n* + 15% of 224 = 258); and it is the final sample size.

### Sampling technique

Clinical medical and other graduating health science students at the verge of completion of their courses during the study year and who were also attending clinical practices in different departments of AMGH or any other hospitals were stratified, based on academic years (for medicine students) and departments (for other graduating class students). A proportional allocation sampling technique was followed for each stratum; as clinical-I, clinical-II, medical intern, graduating class of anesthesia, medical laboratory, midwifery, nursing, radiology and health officer; also a systematic sampling technique was used to recruit the rest. Probability proportional to size /PPS (proportional allocation technique)^11^ was obtained by using the formula,

$$ni=\frac{Ni}{N}*n$$

where: **ni** = Required sample size of students from each ith stratum; **Ni** = Number of medical students in the ith stratum; *n*** = **Over all sample size; ***N*** = Total number of students in the ith stratum.

Absolute proportions were found in anesthesia, 4.7% (*n* = 12), clinical – I medical students, 13.2% (*n* = 34), clinical—II medical students, 13.9% (*n* = 36), medical interns, 25.2% (*n* = 65), health officers, 10.5% (*n* = 27), medical laboratory science, 8.5% (*n* = 22), midwifery, 8.9% (*n* = 23), nursing, 10.8% (*n* = 28) and radiology, 4.3% (*n* = 11).

### Collection methods

#### Data collection tool and procedure

A pre-tested structured questionnaire with three sections was used to collect the data; it is formulated after an extensive literature survey. The first section consisted of socio-demographic questions, the second part comprised clinical data and the third section corresponded questions linked to behavioral peculiarities. Informed consents were sought for all participants prior to study. Socio-demographic and other related information were accumulated by the data collector through a face-to-face interview.

#### Sample collection, transportation, processing and analysis

Nasal samples were collected by inserting a sterile cotton swab (moistened with sterile normal saline prior to sampling, to avoid discomfort) into both nostrils^9^. A single specimen was obtained from each participant from both anterior nares consecutively, using the same swab and it was then placed in a sterile normal saline, transported to the Microbiology and Parasitology Laboratory, Department of Medical Laboratory Sciences, using a cold chain within an hour of collection.

#### Isolation and identification of S. aureus

All the samples were processed immediately to avoid any possible contamination. Each sample was directly inoculated into mannitol salt agar (Oxoid, Hampshire, UK). The inoculated plates were then incubated for 24 h at 37 °C, and the yellowish colonies obtained were subsequently subjected to species identification and confirmation. Morphological, physiological and biochemical characteristics (positive catalase and coagulase tests), of bacteria were ascertained by adopting standard laboratory methods including Gram staining and examination of morphology on different media such as DNase and blood agar^12^. Corresponding American Type Culture Collection (ATCC) strains were utilized as references.

#### Detection of MRSA

Identification of MRSA was performed in accordance with the criteria set by Clinical Laboratory Standard Institute (CLSI), using cefoxitin disk diffusion assay. Bacterial suspension (5 ml) of 0.5 McFarland (1 × 10^8^ CFU/ml) was prepared and swabbed into Mueller Hinton agar (MHA) (Hi-media, India), and cefoxitin disk were placed. After incubation for 24 h at 37 °C, the zone of inhibition was measured. Strains showing zone of inhibition ≤ 21 mm were extrapolated as MRSA^13^.

#### Antimicrobial susceptibility testing

Antibiotic susceptibility profiling of all isolates of MRSA were achieved by Kirby–Bauer disk diffusion technique according to the criteria set by CLSI^13^. Inoculums equivalent to the opacity of 0.5 McFarland standards were prepared and swabbed over MHA surface; exposed to a concentration gradient of antibiotic, and then incubated face up at 37 °C for 24 h. Diameter of zones of inhibition were measured to the nearest millimeter and categorized as sensitive, intermediate, and resistant according to the table described in CLSI. Following antibiotics were used to examine the susceptibility profiles of MRSA, viz., penicillin G (10 units), ciprofloxacin (5 μg), clindamycin (2 μg), gentamicin (10 μg), erythromycin (15 μg), chloramphenicol (30 μg), ampicillin (10 μg), ceftriaxone (30 μg), tetracycline (30 μg) and trimethoprim-sulfamethoxazole (1.25/25.75 μg) and cefoxitin (30 μg)^13^. In addition, inducible clindamycin resistance was also analyzed by disk diffusion using D-zone test. Erythromycin disk (15 μg) was placed at a distance of 15–26 mm (edge to edge) from a clindamycin disk (2 μg) on MHA. After overnight incubation, plates were examined for the formation of flattened zone of inhibition adjacent to the erythromycin disk. A truncated clindamycin zone of inhibition (D-shape) indicated inducible resistance; this method is applied only to organisms which were resistant to erythromycin and susceptible or intermediate to clindamycin^13^.

#### Detection of biofilm

Micro-titer plate assay was used for the detection of biofilm forming MRSA isolates in this study^14^. Well standardized bacterial suspensions were prepared in sterile normal saline from pure colonies and adjusted to a 0.5 McFarland turbidity standard. Standardized bacterial suspensions were diluted with tryptic soy broth (TSB), supplemented with 1% glucose to a final volume of 200 µl per well. This experiment was performed for each isolate in triplicate and incubated at 37 °C for 24 h in flat-bottomed polystyrene micro-titer plates, the contents of the wells were then removed and washed with 300 μl (0.3 ml) of phosphate buffered saline (PBS). Finally, the wells were fixed with 150 μl of 99% methanol for 30 min and stained with crystal violet (0.1% w/v) for 15 min. Excess stain was rinsed off with distilled water. After drying, the wells were treated with 150μL of 95% ethanol for 30 min at room temperature to solubilize the dried crystal violet stain adhered to the biofilm. Optical densities (OD) were then determined by an automated micro ELISA reader at a wavelength of 570 nm. These OD values were considered as an index of bacterial adhesion and biofilm formation. Six wells (A1 to A6) of micro-titer plates were used as negative controls which contained only TSB + 1% glucose without bacteria. Average of three wells used for each bacterial isolate is considered as the actual OD since each of them was added to three wells. The cut-off value of optical density (ODc) was calculated and defined as three standard deviations above the mean OD of the negative control.

$${\text{ODc}} = {\text{Average}}\;{\text{OD}}\;{\text{of}}\;{\text{Negative}}\;{\text{controls}} + 3 \times {\text{Standard}}\;{\text{deviation}}\;{\text{of}}\;{\text{negative}}\;{\text{controls}}$$

Isolates were classified as follows: as described elsewhere^14^.

1. Bacterial OD ≤ ODc = non-biofilm former;
2. OD > ODc, but ≤ 2 ODc = weak biofilm former;
3. OD > 2 ODc, but ≤ 4 ODc = moderate biofilm former and
4. OD > 4ODc = strong biofilm former

#### Quality control

A pre-test was done on 5% of the total sample size before one week of the actual study in Wolita Sodo University to validate the variables and data collection tools were modified accordingly. Standard quality measures were implemented throughout the entire process of data collection and the laboratory works and also the completeness, accuracy, clarity, and consistency of data were checked. Standard Operating Procedures (in-house SOP manual) for each operation were strictly followed. All culture media were prepared following the instructions of manufacturers and sterility was tested by incubating 5% of each batch at 35–37 °C overnight, for the evaluation of any possible contamination. Moreover, positive control (standard) strains of *S. aureus*, (ATCC 25923) were used as the quality controls (reference) for biochemical tests and agar plates including MHA with vancomycin disk; this ensured the testing performance, i.e., the potency of the disk, as per CLSI 2019 guidelines.

### Data analysis

Data were checked, cleaned and coded for its completeness and entered into the Epi data version 4.6.0.2 software, and analyzed by the Statistical Package for Social Sciences (SPSS) version 25. Descriptive statistics including frequency, mean and percentages were used. Binary logistic regression model was used to analyze the association among dependent and independent variables. Those variables with *p* value < 0.25 in bivariable analysis were considered as candidates for further multivariable analysis; *p* value ≤ 0.05 was considered as statistically significant. Adjusted odds ratio (AOR) and 95% confidence interval (CI) were used to determine the strength of association among variables.

## Data Availability

All relevant data are within the manuscript.
